# Diagnostic performance of digital tomosynthesis for postoperative assessment of acetabular fractures and pelvic ring injuries

**DOI:** 10.3389/fsurg.2024.1461144

**Published:** 2024-10-25

**Authors:** Atticus Coscia, Eric Schweppe, Jason Anari, Bruce Kneeland, Annamarie Horan, Samir Mehta, Jaimo Ahn

**Affiliations:** ^1^ Department of Orthopaedic Surgery, University of Michigan, Ann Arbor, MI, United States; ^2^ Department of Orthopaedic Surgery, University of Pennsylvania, Philadelphia, PA, United States

**Keywords:** digital tomosynthesis, postoperative computed tomography, pelvic ring, acetabulum, orthopaedic imaging

## Abstract

**Introduction:**

Digital tomosynthesis (DTS) has broad non-orthopaedic applications and has seen limited use within orthopaedics for imaging of the wrist. The utility of DTS for assessing reduction and hardware placement following operative treatment of pelvic ring injuries and acetabular fractures has not previously been investigated.

**Methods:**

All operatively treated acetabular fractures and pelvic ring injuries that underwent surgical fixation within a one-year time span received both a computed tomography (CT) scan and a DTS scan as part of their routine postoperative imaging workup. Three orthopaedic traumatologists independently reviewed the images for face-value clinical utility. Radimetrics and PCXMC software were utilized on a subset of the study population's DTS and CT studies to calculate the effective and organ radiation doses delivered.

**Results:**

52 patients (22 acetabular fractures, 22 pelvic ring injuries, 7 pelvic ring and acetabular fractures, 2 femoral head & acetabular fractures) were included. DTS was considered adequate to assess reduction 83% of the time, hardware position 83% of the time, and sufficient to replace the CT scan 67% of the time. DTS was associated with an 8.3 times lower effective radiation dose than CT. All organ doses were lower for DTS than CT.

**Discussion:**

DTS appears to have face-value clinical utility for assessing reduction and hardware position following surgical treatment of acetabular fractures and pelvic ring injuries. Given that DTS is associated with significantly lower radiation doses to patients, further study utilizing more objective criteria is warranted.

## Introduction

Displaced acetabular fractures and unstable pelvic ring injuries often require surgical intervention to improve functional outcomes (1, 2). Malreduction and misplaced implants have each been shown to contribute to poor clinical results following surgical fixation (2–5). Intraoperative fluoroscopy and postoperative plain radiographs are traditionally used to assess reduction quality and implant position (6). However, these imaging modalities provide inferior diagnostic sensitivity compared to computed tomography (CT) (5, 7–11). The routine use of postoperative CT imaging for all surgically treated acetabular fractures and pelvic ring injuries remains controversial. Some authors recommend judicious use of postoperative CT scans in select patients with complex injury patterns, demonstrating low rates of revision indicated by postoperative imaging results (7, 9, 12, 13). Meanwhile, others have found higher rates of revision or have been unable to determine risk factors for reoperation and suggest that postoperative CT scans should be obtained routinely (10, 11, 14). Despite these differing recommendations, the fact remains that across previous studies, the majority of patients will not require revision surgery based upon their postoperative CT results. It remains unknown if there are alternative imaging options which would allow adequate assessment of reduction and implant position while reducing cost and radiation exposure.

Digital tomosynthesis (DTS) represents a potential alternative postoperative imaging option. DTS involves tomographic reconstruction of linear radiography images obtained using a limited sweep of between 30° and 60° (compared to 360° in CT) (15–17). DTS is a functional compromise between CT and plain radiography because this technique generates CT-like cross sectional imaging in a single plane at significantly reduced radiation dosage and cost compared to CT (15–17). DTS is routinely used in various applications including mammography and pulmonary nodule surveillance (18, 19). DTS use in orthopaedic indications has remained comparatively limited. To date, DTS has most frequently been utilized in orthopaedics for imaging of the wrist. Several series have been published establishing the superior diagnostic accuracy of DTS over plain radiography to detect fractures and bone erosions (15, 17, 20, 21). DTS has also shown some promise for imaging of total hip and knee arthroplasty components and one case series explored its application in an orthopaedic trauma population (22–26). The utility of DTS for assessing fracture reduction and implant placement following surgical fixation of acetabular fractures and pelvic ring injuries has, to our knowledge, not previously been investigated.

The purpose of this study was to prospectively investigate the utility of DTS imaging for assessment of hardware position and fracture reduction in operatively treated patients with pelvic ring injuries and acetabular fractures. We hypothesized that DTS would provide adequate visualization to replace CT for assessment of hardware placement and fracture reduction for most patients.

## Methods

After institutional review board approval, all surgically treated acetabular fractures and pelvic ring injuries treated at our urban level I trauma center were prospectively enrolled from 1/1/2016–1/1/2017. The standard of care in the University of Pennsylvania Health system includes the following imaging studies when evaluating a patient with a pelvic ring or acetabular injury: Preoperatively, all patients receive an AP pelvis digital radiograph and a CT scan of the abdomen and pelvis. 3-D reconstructions (including inlet, outlet, obturator oblique, and iliac oblique) are rendered from the CT scan. Intra-operative fluoroscopy aids the assessment of reduction and application of hardware. Post-operatively, an AP pelvis digital radiograph and a CT scan of the pelvis with digitally rendered inlet, outlet, iliac oblique, and obturator oblique views are obtained. The study design follows standard of care substituting DTS post-operatively instead of an AP pelvis digital radiograph. Of note, the AP pelvis digital radiograph is reconstructed as part of the DTS sweep.

DTS images were collected on a Healthcare Discovery XR656 or XR656 Plus (General Electric, Fairfield, Connecticut) systems. Multiple tomographic “slices” in the coronal plane separated by 2 mm slice intervals were generated using a single sweep in the superior-to-inferior direction. Three fellowship-trained orthopaedic traumatologists, each with experience treating patients with pelvic ring and acetabular injuries, reviewed the images independently for face-value clinical utility. Traumatologists responded either yes or no to the following questions: 1- “Can you assess the fracture pattern of the pelvic ring or acetabular injury?” 2- “Can you assess the reduction of the pelvic ring or acetabular fracture?”, 3- “Can you assess hardware placement?” and 4- “Do you feel the DTS provides adequate information to replace the CT scan?”

A subset of four patients was selected for the purpose of calculating the organ dose and effective dose to patients from DTS as compared to CT. Organ and effective doses to patients secondary to CT were calculated using Radimetrics (Bayer, Whippany, NJ). Organ and effective doses to patients secondary to DTS were calculated using PCXMC (Stuk, Vantaa, Finland). Organ and effective doses were calculated according to the International Commission on Radiological Protection publication 103 recommendations (27). Effective doses are reported in units of milli-Sievert (mSv). Organ doses are reported in units of milligray (mGy). Table 12−1D from the BEIR VII report was used to calculate lifetime attributable risk of the radiation doses associated with DTS and CT (28).

### Statistical analysis

Fixation tactic was coded such that 0 = open reduction internal fixation (ORIF) and 1 = closed reduction and percutaneous fixation (CRPP). Sex was coded such that 0 = Male and 1 = Female. Dependent variables (questions regarding fracture pattern, reduction, hardware safety, and replacing CT) were coded such that a value of 3 reflected that all reading surgeons answered “yes” when queried regarding this variable, while 0 reflected that all of the reading surgeons answered “no” when queried.

Data were prescreened to check for outliers and missing data. Descriptive analyses and correlations were conducted for all primary variables. Assumptions for multiple regression (i.e., linearity, homoscedasticity, independence) were evaluated. Injury type and age were centered to aid in interpretability. Multivariate linear regression was conducted using the PROC REG statement in SAS 9.4 to test study hypotheses (SAS Institute, Inc., Cary, NC, Copyright © 2016). Separate models were estimated to test the association between fixation tactic and each dependent variable, for a total of four models. Age, sex, and injury type were included as covariates in all models.

## Results

**Figure 1:**
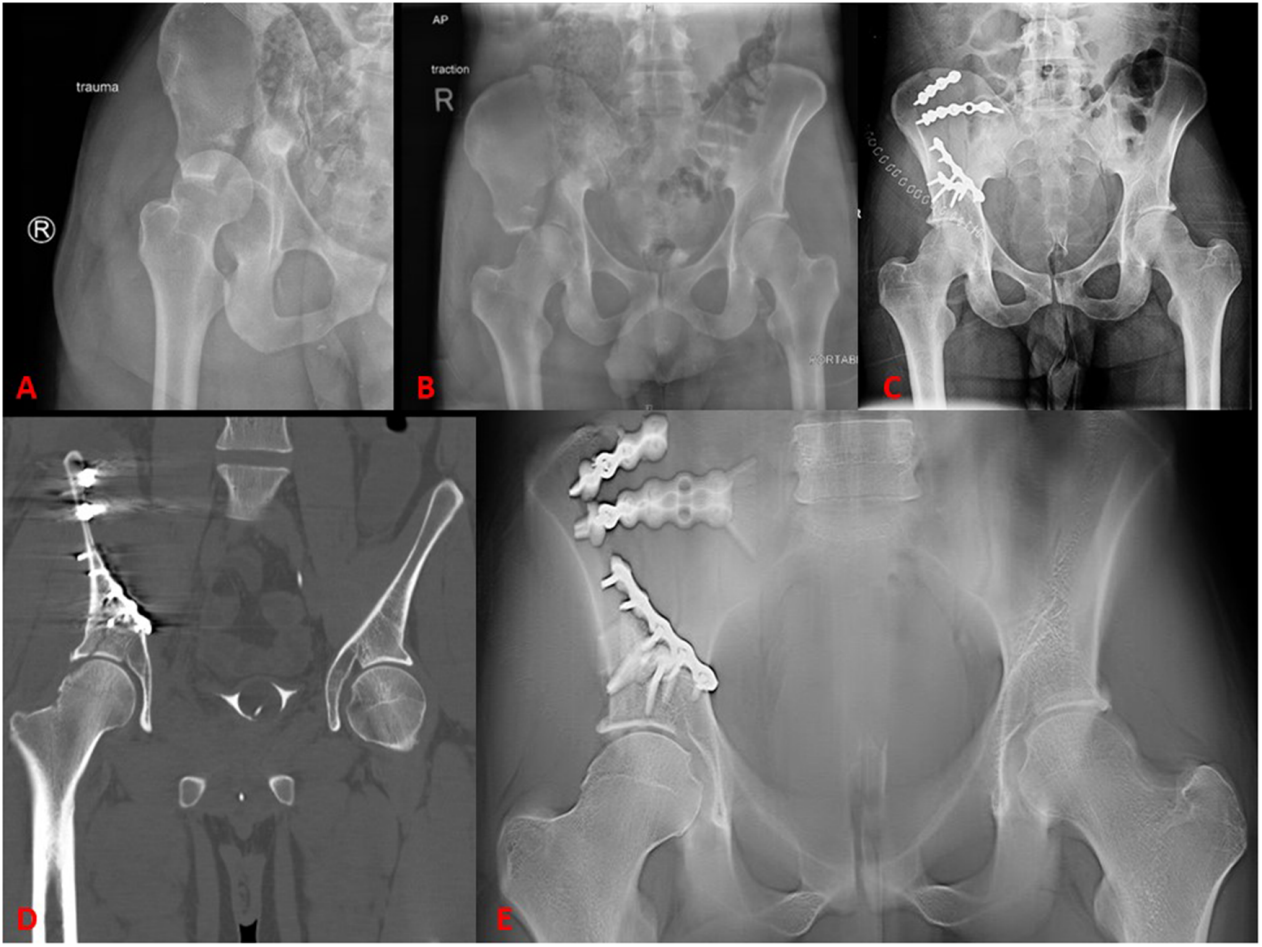
AP digital radiograph of the right right hip of a 28-year-old male **(A)** with a R acetabular fracture and hip dislocation. AP pelvis digital radiograph of the same patient **(B)** following closed reduction. Post-operative AP pelvis digital radiograph **(C)**, representative coronal slice from postoperative computed tomography scan **(D)** representative coronal slice from digital tomogram **(E)**.

A total of fifty-three participants were considered for data analysis. *N* = 1 participant did not have fracture fixation; therefore, this participant was excluded from further data analysis. Therefore, *N* = 52 participants are included in all analyses. Figure 1 depicts injury and postoperative radiographs, postoperative CT images, and postoperative digital tomograms for a patient who sustained a right acetabular fracture and hip dislocation.

### Descriptive statistics

Descriptive statistics are presented in Table 1. Participants ranged in age from 18- to 95-years-old. On average, participants were 47 years old (standard deviation age = 19.03) and the sample had slightly more males (52.83%). With respect to injury type, most of the sample presented with acetabular fractures (*n* = 22) or pelvic ring injuries (*n* = 22). *N* = 7 and *n* = 2 patients presented with pelvic ring & acetabular fractures, and *n* = 2 femoral head fractures and acetabular fractures. Regarding fracture fixation, slightly more patients received CRPP (*n* = 28) than ORIF (*n* = 24). The three traumatologists found the DTS imaging adequate to assess the fracture pattern 26% of the time, reduction of the fracture or injury 83% of the time, safety of hardware position 83% of the time and felt that the imaging studies were sufficient to replace a CT scan post-operatively 67% of the time.

**Table 1:** Bivariate correlations and descriptive statistics for main study variables.

Variable	Age	Sex	Fixation tactic	Injury type	Fracture pattern	Reduction	Hardware safety	Replace CT
Age								
Sex	0.3**							
Fixation tactic	0.2	0.1						
Injury type	0	0.1	0.1					
Fracture pattern	0.1	−0.2	0.2	0				
Reduction	0.1	−0.2	0.3*	0.1	**0** **.** **4**			
Hardware safety	−0.3*	−0.2	−0.1	−0.1	0.3*	**0** **.** **5**		
Replace CT	−0.1	−0.2	0.2	−0.1	**0** **.** **4**	**0** **.** **6**	**0** **.** **6**	
Mean	47	–	–	–	0.8	2.5	2.5	2
Standard deviation	19	–	–	–	0.8	0.7	0.7	0.8

* *p = 0.06.* **p < .05; Bolded = *p* < .001. Dependent variables were coded such that a value of 3 reflected that all reading surgeons answered “yes” when queried regarding this variable, while 0 reflected that none of the reading surgeons answered “yes” when queried.

With respect to zero-order correlations, in general, all dependent variables were strongly correlated such that high scoring on one dependent variable was strongly correlated with higher scoring on all others (see Table 1). For example, when a high number of physicians rated fracture pattern as yes, they also rated reduction, hardware safety, and replace CT as yes. Regarding covariates, age was correlated with sex (*r* = 0.34, *p* < .05) and marginally correlated with hardware safety (*r* = −0.26, *p* = .06), such that older patients were more likely to be female and less likely to have their hardware safety rated as “yes”. With respect to the primary independent variable of interest, fracture fixation was marginally correlated with reduction (*r* = 0.26, *p* = .06), such that patients who received CRPP received yes ratings on the reduction query more frequently.

### Multivariate linear regression

Results from multivariate linear regression are reported in Table 2.

**Table 2:** Multivariate linear regression results.

Variable	Fracture pattern			Reduction			Hardware safety			Replace CT scan		
	β	S.E.	*t* value	β	S.E.	*t* value	β	S.E.	*t* value	β	S.E.	*t* value
Intercept	0.7	0.2	3.5**	2.5	0.2	**15** **.** **8**	2.6	0.2	**15**	2.1	0.2	**11** **.** **1**
Fracture fixation	0.4	0.3	1.5	0.4	0.2	1.9	0	0.2	−0.1	0.3	0.2	1.5
Age	0	0	0.5	0	0	0.9	0	0	0	0	−0.2	3.2
Sex	−0.3	0.3	−1.3	−0.4	0.2	−2.2*	−0.2	0.2	−0.4	0.2	−1.6	−1.3
Injury type	0	0.1	−0.2	0.1	0.0	0.9	0	0.1	−0.4	−0.1	0.1	−0.5

Sex was coded such that 0 = male, 1 = female. Dependent variables were coded such that a value of 3 reflected that all reading surgeons answered “yes” when queried regarding this variable, while 0 reflected that none of the reading surgeons answered “yes” when queried. Bolded values = *p* < .001, ** = *p* < .01, * = *p* < .05.

#### Fracture pattern

Fracture fixation was not associated with the number of yes ratings from physicians on fracture pattern (*B* = 0.36, *p* = .15). No covariates significantly predicted the number of yes ratings from physicians on fracture pattern.

#### Reduction

Fracture fixation was significantly associated with the number of yes ratings from physicians on reduction at the trend level (*B* = 0.35, *p* = .06). The direction of this effect suggested that patients who received CRPP had a higher number of physicians rate yes on reduction. With respect to covariates, sex was significantly associated with the number of yes ratings, such that males received a higher number of physicians rate yes on reduction. No other associations were significant.

#### Hardware safety

Fracture fixation was not associated with the number of yes ratings from physicians on hardware safety (*B* = −0.02, *p* = .93). No covariates significantly predicted the number of yes ratings from physicians on hardware safety.

#### Replace CT scan

Fracture fixation was not associated with the number of yes ratings from physicians on replace CT scan (*B* = 0.32, *p* = .14). No covariates significantly predicted the number of yes ratings from physicians on replace CT scan.

### Radiation dose for DTS and CT

On average, the effective dose to patients from DTS was 8.3 times lower than the effective dose from CT (Table 3). The average organ doses were also lower for DTS as compared to CT. The organ doses are displayed in Table 3 listed and are organized based upon whether the specified organ was in- or out of the field of view. Organs that were out of the field of view had intrinsically lower doses for both DTS and CT. However, organs that were out of the field of view had a relatively higher ratio of radiation exposure during CT relative to DTS.

**Table 3:** Effective and organ doses for DTS and CT.

Patient	DTS (mSv)	CT (mSV)	Ratio
1	2.2	8.2	3.7
2	2.0	23	12
3	1.4	20	14
4	1.6	7.7	4.8
Average	1.8	15	8.3
Organ	DTS (mGy)	CT (mGy)	Ratio
In field of view			
Bone marrow	0.5	11	22
Muscle	1.4	13	9.3
Skeleton	1.0	13	13
Large intestine	2.4	30	13
Adrenals	0	1.0	
Small intestines	1.8	30	17
Urinary bladder	9.1	37	4.1
Testicles	15	34	2.3
Skin	1.2	10	8.3
Out of field of view			
Brain	0	0	
Gall bladder	0.2	11	55
Heart	0	0.3	
Kidneys	0.1	8.2	82
Liver	0	4.0	
Lungs	0	0.2	
Muscle	1.4	12	8.6
Oesophagus	0	0.4	
Pancreas	0	2.4	
Salivary glands	0	0	
Spleen	0	2.6	
Stomach	0.1	6.0	60
Thymus	0	0	
Thyroid	0	0	

mSV, milli-Sievert; mGy, milligray.

## Discussion

Operative intervention is frequently indicated for the treatment of displaced acetabular fractures and unstable pelvic ring injuries. Digital radiography and CT are the standard of care for initial injury assessment and preoperative planning, while fluoroscopy is utilized intraoperatively to guide reduction and hardware placement. The optimal postoperative imaging protocol, specifically the utility of postoperative CT, remains controversial. Some feel that postoperative CT should be routinely ordered for all patients (10, 11, 14). Others recommend that CTs be obtained more judiciously, considering increased cost and radiation dose to be prohibitive given the overall low diagnostic yield (7, 9, 12, 13). DTS has shown utility in non-orthopaedic applications, diagnosis of fractures of the wrist, and for assessment of hip and knee arthroplasty. It remains unknown if DTS represents a viable alternative to CT for assessment of reduction and hardware placement following operative treatment of acetabular fractures and pelvic ring injuries.

Our results suggest that DTS has potential as a clinically useful imaging modality in this role. DTS studies were rated as adequate for assessment of reduction and hardware position 83% of the time and sufficient to replace a CT study 67% of the time. These results illustrate the potential for DTS to significantly decrease postoperative cost and radiation dose by reducing the need for routine postoperative CT. This is the first study, to our knowledge, to investigate the utility of DTS in the assessment of pelvic ring injuries and acetabular fractures and thus comparison to previous literature is limited. However, our results show good agreement with previous data investigating the performance of DTS for assessment of the wrist. Perloff and colleagues found that DTS provided satisfactory diagnostic information and obviated the need for advanced imaging to rule out the presence of occult scaphoid fracture in 69% of patients (15). Ottenin and colleagues illustrated that DTS had adequate sensitivity and specificity to successfully diagnose wrist fractures for most patients. These authors suggested that, when used as a complement to radiography, DTS could eliminate the need for CT for certain patients (21). Although these results are promising, it is important to consider that a common theme between our results and previous work is that DTS provided adequate diagnostic information to replace CT for many, but not all, patients.

Further analysis offered some preliminary insight into the patients and injury patterns for which DTS may be most useful. Correlation data illustrated that all dependent variables were strongly associated, such that when a greater number of physicians rated one dependent variable (e.g., fracture pattern) as “yes”, they also rated all other dependent variables (e.g., reduction, hardware safety, replace CT) as “yes” also. This finding suggests that DTS consistently provided diagnostic information across dependent variables and was likely adequate to replace a CT scan for a subgroup of the study population. Correlation analysis also indicated that age was associated with sex and marginally correlated with hardware safety, such that older patients were more likely to be female and less likely to have a high rating on the outcome of hardware safety. Multivariable regression analysis showed that males were significantly more likely to have a higher score on reduction while fracture fixation via CRPP was marginally associated with higher scores on reduction. Taken together, these results suggest that DTS may be most useful for postoperative assessment in younger patients with fracture patterns that are amenable to CRPP.

These findings are not surprising when contextualized within the epidemiology of acetabular fractures and pelvic ring injuries and the limitations of DTS are considered. Acetabular fractures and pelvic ring injuries occur most commonly in female patients in the setting of insufficiency fractures and advanced age (e.g., unimodal distribution) (29). Meanwhile, in males these injury patterns generally follow a bimodal distribution, presenting in younger patients in the setting of high energy trauma, as well as older patients in the setting of fragility fracture (29). Therefore, it follows that older patients in the current study were more likely to be female, who sustained fragility fractures in the setting of significant osteoporosis. Severe bony demineralization compounded by complex three-dimensional anatomy and overlying bowel gas present unique challenges for adequate visualization of pelvic and acetabular fragility fractures (30). Indeed, even in young, healthy patients with normal bone stock, pelvic ring injuries and acetabular fractures are generally complex from both a diagnostic and therapeutic standpoint due to associated parenchymal injuries, considerable bleeding, and concomitant skeletal injuries (31). DTS may not be best suited for this role for a variety of reasons. The primary limitations of DTS include the inability to acquire axial images, reformat data along any plane, or create surface-rendered images. These are all functionalities that are possible with CT. As such, CT provides more extensive visualization of acetabular fractures and pelvic ring injuries in multiple planes, allowing for more thorough assessment. This also likely relates to why DTS showed better results for fractures fixed via CRPP. Injuries treated exclusively via CRPP are typically less complex and are less likely to have significant articular involvement (32). As such, it is possible that the reduction and hardware placement for these injuries was more easily assessed on cross sectional imaging obtained in a single plane (i.e., DTS) and without the inclusion of axial cuts. Supplementary File S1 presents an algorithm for when DTS may be most clinically useful, based upon these preliminary data.

Although DTS does not provide axial images or allow for the reformatting of data along any plane, these limitations are offset by the primary advantage of DTS: decreased radiation dose. Patient safety is paramount, and physicians are obligated to investigate any opportunity to improve the standard of care and patient safety. Although CT scans deliver clear cut images in multiple planes, the amount of radiation a patient receives compared to digital radiography is increased anywhere from 5 to 1,000-fold, depending on variables such as the anatomy of interest, scan type, and patient body habitus (33, 34). Previous work has illustrated that DTS radiation dosing is nearly a factor of 10 lower than CT, as measured by dosimeters implanted in an anthropomorphic phantom (33). Meanwhile, the added affective dose from tomosynthesis to the compliment radiography study has been estimated to add only 2–5× the radiation dosage, significantly lower than CT (35). Our findings show good agreement with previous data, illustrating an overall 8.3 times lower effective radiation dose with DTS as compared to CT. All organ doses were also smaller for DTS as compared to CT. Testicular dose, which was only 2.3 times less than CT, had the largest impact on DTS effective dosing. This was due to the fact that the testicles are external to the body and located anterior (e.g., closest to the x-ray tube). Meanwhile, during a CT the testicles are shielded by the legs laterally. Although organ dosing was much smaller for organs that were both in- and out of field of view the ratio of radiation dose for out of field organs (e.g., CT organ dose relative to DTS organ dose) was much larger due to the greater amount of scatter associated with CT.

The reduced radiation dosages calculated for DTS were associated with corresponding reductions in cancer-induced death. Overall risk of cancer-induced death associated with a DTS study (0.008185%) was 7.2 times lower compared to CT (0.059375%). This findings show good agreement with previous work by Wylie and colleagues who found that two CT scan doses were associated with a 17.5 times higher risk of cancer in males and 16.1 times higher risk of cancer in females (i.e., roughly double) for patients who received pre- and postoperative pelvic CT (i.e., for two CTs vs. one in the current study) as compared to those who received radiographs only (36). Based on the most recently published epidemiological data for acetabular fractures and pelvic ring injuries in the United States, if all postoperative CT studies were replaced with DTS, this would result in the prevention of approximately 7,500 cancer-induced deaths per year (assuming all acetabular fractures and pelvic ring injuries were surgically treated and postoperative CT imaging would have otherwise been obtained for all patients) (37–39).

This study is not without its methodological limitations. In particular, the traumatologists evaluating the cases were the treating surgeons so their knowledge of the fracture anatomy is not entirely blinded. In addition, the variables measured were based on subjective utility to the surgeon and lacked an objective or reference standard. The current data were collected for the purposes of preliminary analysis and is intended to provide a foundation and compelling basis for the further study of more objective measures. As we continue data collection, validity and reliability will be further evaluated.

In conclusion, our data indicates that DTS has face-value utility to the orthopaedic surgeon in the post-operative evaluation of pelvic ring and acetabular injuries. Taken together our results support the consensus of previous authors who have investigated the application of DTS in orthopaedics: DTS offers a compromise between digital radiography and CT. Given its cross-sectional nature, DTS offers diagnostic information superior to digital radiography. DTS does not provide imaging in multiple planes or axial views, and thus does not provide as extensive visualization as CT. However, DTS is associated with significantly less cost and radiation exposure. Further work is warranted to better characterize the diagnostic utility of DTS for the postoperative assessment of acetabular fractures and pelvic ring injuries. Specifically, more detailed analysis with a larger number of patients as well as participating surgeons and radiologists should be undertaken to more clearly delineate when DTS can successfully function as an alternative modality to CT. It is possible that DTS can function as an alternative postoperative imaging modality for pelvic ring injuries and acetabular fractures, with CT ordered on an as needed basis, leading to significant reductions in cost and radiation exposure.

## Data Availability

The raw data supporting the conclusions of this article will be made available by the authors, without undue reservation.

## Supplementary material

Supplementary File S1 Algorithm illustrating when digital tomosynthesis may be considered as an alternative to computed tomography for postoperative assessment of acetabular fractures and pelvic ring injuries.
