# Supplementary figures and images for: Diagnostic performance of digital tomosynthesis for postoperative assessment of acetabular fractures and pelvic ring injuries
Source: Front Surg. 2024 Oct 25;11:1461144. doi: 10.3389/fsurg.2024.1461144 (PMC11543565; doi:10.3389/fsurg.2024.1461144)

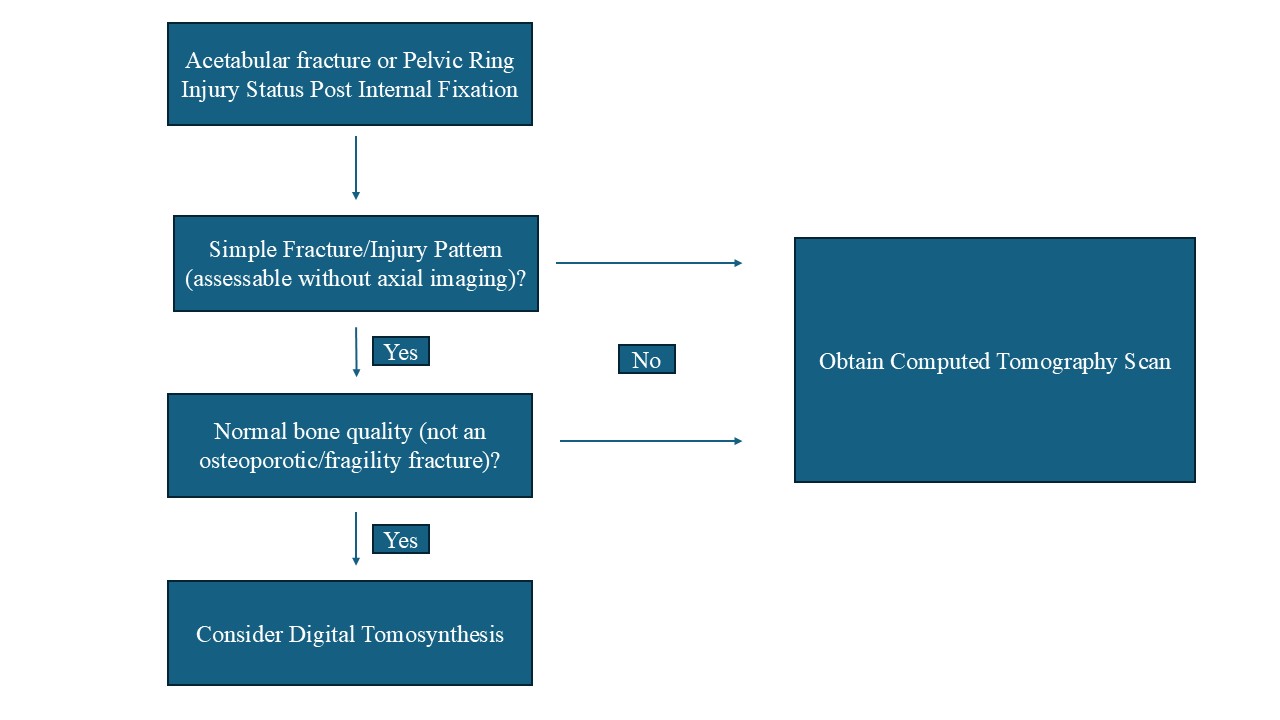

Supplement: Supplementary File S1 — Algorithm illustrating when digital tomosynthesis may be considered as an alternative to computed tomography for postoperative assessment of acetabular fractures and pelvic ring injuries. [file Image1.jpeg]
